# Community-based peer education as a tool for the prevention of breast cancer: a narrative review

**DOI:** 10.3332/ecancer.2026.2108

**Published:** 2026-04-07

**Authors:** Rachael O Oduyemi, Chizoma Millicent Ndikom, Gloria Oluwakorede Alao, Iyanuoluwa O Ojo, Faith Ayomide Ajayi, Damilola Ajibade, Abdullahi Suleiman, Hameedah Ayomide Gbadamosi, Oluwadamilare Akingbade

**Affiliations:** 1 Faculty of Nursing Sciences, Chrisland University, Abeokuta, 110104, Ogun State, Nigeria; 2 Faculty of Nursing, College of Medicine, University of Ibadan, Ibadan, 200001, Oyo State, Nigeria; 3 Institute of Nursing Research, Osogbo, 232111, Osun State, Nigeria; 4 Nursiscope Mentorship Academy, Nursiscope Inc., Edmonton, T5Y OW4, Canada; 5 Ahmadu Bello University, Zaria, 810001, Nigeria; 6 Chrisland University, Medical Centre, Abeokuta, 110104, Ogun State, Nigeria; 7 Bayero University Kano, Kano, 7000001, Kano State, Nigeria; 8 Olabisi Onabanjo University Teaching Hospital, Sagamu, 121101, Ogun State, Nigeria; 9 School of Nursing, Dalhousie University, Halifax, B3H 4R2, Canada; a https://orcid.org/0000-0003-2451-7277; b https://orcid.org/0000-0002-4036-156X; c https://orcid.org/0009-0009-9547-3831; d https://orcid.org/0000-0002-2132-2987; e https://orcid.org/0009-0006-9928-4294; f https://orcid.org/0009-0004-7141-8030; g https://orcid.org/0009-0002-0137-6977; h https://orcid.org/0009-0002-5771-0559; i https://orcid.org/0000-0003-1049-668X

**Keywords:** breast, cancer, peer-based education, community

## Abstract

**Background:**

Breast cancer remains a major threat to women, especially in low-resource settings like Nigeria, where awareness and screening are limited. Peer education may offer a practical way to improve early detection and prevention in these communities.

**Objective:**

To examine how peer education supports breast cancer prevention in communities by exploring its delivery methods, impact on knowledge and behaviour and the cultural and practical factors that shape its effectiveness.

**Methods:**

A narrative review was conducted to explore the role of community-based peer education in breast cancer prevention. Relevant studies published between 2010 and 2025 were identified through a systematic search of six databases (CINAHL, PubMed, Web of Science, Embase, Medline and APA PsycINFO) using specific keywords. After screening and applying inclusion and exclusion criteria, data were extracted from selected studies and thematically analysed to identify patterns, benefits and challenges of peer education interventions.

**Result:**

Out of 818 studies initially retrieved, only 18 met the eligibility criteria and were reviewed, covering diverse locations and participant groups, mostly from high-income countries. The studies showed that peer education delivered through various methods and tailored to cultural contexts significantly improved breast cancer awareness, screening behaviours and community engagement, though challenges like role confusion, cultural barriers and logistical issues were also reported.

**Conclusion:**

This review concludes that peer education is an effective, adaptable strategy for promoting breast cancer prevention in community settings, especially among underserved populations.

## Background

Globally, cancer continues to rise at an alarming pace, affecting millions each year and placing an immense burden on individuals, families and healthcare systems. In 2022, the International Agency for Research on Cancer reported nearly 20 million new cancer cases worldwide, resulting in approximately 9.7 million deaths. Lung cancer was the most commonly diagnosed cancer, followed by breast cancer in women [1].

In Nigeria, the number of new cancer cases increased to 127,763, with 79,542 deaths and breast cancer emerged as the most prevalent, accounting for 25.3% of cases [2].

The uptake in Nigeria on the practice of breast self-examination (BSE), clinical breast examination and mammography remains low due to poor awareness, sociocultural beliefs, limited access and inadequate knowledge [3, 4].

Systemic delays within the health system such as fragmented delivery of health services, shortages in the health workforce, inadequate health financing, weak health information systems and limited access to essential medicines and technologies further worsen outcomes, as most Nigerian patients present at late disease stages. For example, a Northern Nigeria study found 99.4% presented with advanced disease [5], while a review reported symptom durations of 8–12 months before diagnosis [6]. These findings underscore the need for scalable interventions targeting awareness and behaviour at the community level.

Community-based peer education is an approach to health education and promotion in which trained community members known as peers educate and train others within the same community to improve attitude, knowledge and health-seeking behaviour to specific issues. Community-based peer education offers a promising solution. Peer-to-peer models leverage trust and shared experiences to deliver culturally relevant health messages. Successfully used in HIV prevention and adolescent health [7], peer education has also improved breast cancer screening uptake, as seen in Minnesota’s Breast Cancer Champions program [8]. However, evidence from Sub-Saharan Africa is limited. In Nigeria, few studies have applied peer-led models to breast cancer prevention. One study showed peer-led training improved BSE practices among students [9, 10], yet little is known about the cultural adaptation or outcomes of such interventions in rural and underserved communities.

This review will provide context-specific insights into how peer-led education can enhance breast cancer prevention in Nigeria. Findings will guide the design and scaling of culturally appropriate, community-driven programs that strengthen early detection and reduce mortality [7, 8, 10].

This review aims to:

1. To examine the structure, strategies and delivery methods of peer education programs used in community settings for breast cancer prevention.
2. To assess the impact of community-based peer education on knowledge, attitudes and screening behaviours related to breast cancer among women.
3. To identify contextual and cultural factors that influence the effectiveness of peer education interventions in rural and underserved communities.
4. To explore the challenges and facilitators associated with implementing peer-led breast cancer prevention programs.
5. To provide evidence-based recommendations for designing, adapting or scaling up peer education models for breast cancer prevention in similar contexts.

## Methods

A narrative review approach was adopted to examine the role of community-based education in breast cancer prevention, as this approach enables a thorough understanding by synthesising a broad spectrum of literature [11]. The study aims to explore and synthesise existing evidence on the role of community-based peer education in the prevention of breast cancer . This narrative review was conducted and reported in accordance with the Scale for the Assessment of Narrative Review Articles (SANRAs). The quality of this narrative review was also assessed using the SANRA, a validated tool designed to improve the methodological rigor and reporting quality of narrative reviews [12]. SANRA consists of six criteria: (1) justification of the article’s importance, (2) clarity of aims, (3) description of the literature search, (4) quality and appropriateness of referencing, (5) scientific reasoning and (6) presentation of relevant data in an organised manner. Each criterion was rated on a 0–2 scale, where 0 indicates low quality, 1 indicates moderate quality and 2 indicates high quality, resulting in a maximum possible score of 12. The current review attained a SANRA score of 12, reflecting clarity of the review objectives, transparent description of the search process, appropriate and consistent referencing, logical synthesis of evidence and structured presentation of findings. The application of SANRA strengthened the overall rigor, transparency and reliability of this narrative review.

### Search strategy

A systematic literature search was conducted to identify relevant studies that discussed the utilisation of community-based peer education in the prevention of breast cancer. Six databases were searched including CINAHL, PubMed, Web of Science, Embase,Medline and APA PsycINFO using keywords including ‘breast cancer,’ ‘breast carcinoma,’ ‘breast neoplasm’, ‘mammary cancer’, ‘Breast Neoplasms’, ‘prevention’, ‘risk reduction’, ‘screening’, ‘screen’, ‘breast self-exam’, ‘clinical breast exam’, ‘health promotion’, ‘Primary Prevention’, ‘Early Detection of Cancer’, ‘Mammography’, ‘Health Promotion’, ‘peer education’, ‘peer-led’, ‘peer to peer’, ‘peer support’, ‘lay health’, ‘worker’, ‘lay educator’, ‘community health volunteer’, ‘peer mentor’, ‘Peer Group/education’, ‘Community Health Workers’, ‘Health Education/methods’, ‘community-based’, ‘community setting’, ‘rural community’, ‘village’, ‘low-resource’, ‘underserved’, ‘grassroots’, ‘population-based’, ‘Nigeria’, ‘sub-Saharan Africa’, ‘Community Health Services’ and ‘Rural Health’. Results were exported to the Covidence software for de-duplication and screening.

#### Interventions and comparisons

The review considered peer education programs aimed at increasing awareness, improving knowledge and enhancing uptake of breast cancer prevention and screening services. Interventions included home visits, group workshops, culturally tailored community education, storytelling, faith-based activities, health fairs and mobile health tools.

#### Inclusion and exclusion criteria

Studies were included in this narrative review if they focused on community-based or peer-led education interventions aimed at the prevention of breast cancer. Eligible studies targeted women of any age group and were conducted in community or rural settings. The review considered quantitative, qualitative and mixed-methods studies that provided relevant data. Only articles published in peer-reviewed journals, written in English and recently published within 2010–2025 were included to ensure the review captured recent developments and trends in the field.

Studies were excluded if they were hospital-based or focused on clinic-led interventions with no community or peer education component. Articles that addressed types of cancer other than breast cancer, or those that were editorials, reviews, commentaries or opinion pieces or non peer-reviewed studies were also excluded. In addition, studies not published in English or not available in full-text format were excluded from the review. Decisions for data inclusion and exclusion were documented using the PRISMA chart.

#### Data screening

Title and abstract screenings of relevant papers were conducted according to the established criteria, focusing on the role of community-based peer education in the prevention of breast cancer.

#### Data extraction

Three authors independently extracted data from the selected articles using a data extraction form. This form was developed and pilot-tested on three articles, after which no further modifications were necessary. Extracted information included authors, publication year, study objectives, role of community-based education on breast cancer prevention, benefits, challenges and limitations of community-based education, as well as recommendations for their implementation. To ensure accuracy and completeness, a fourth reviewer verified the extracted data. The collected data were then synthesised thematically to identify patterns, trends and gaps within the literature.

## Results

### Search results

**Figure 1.**
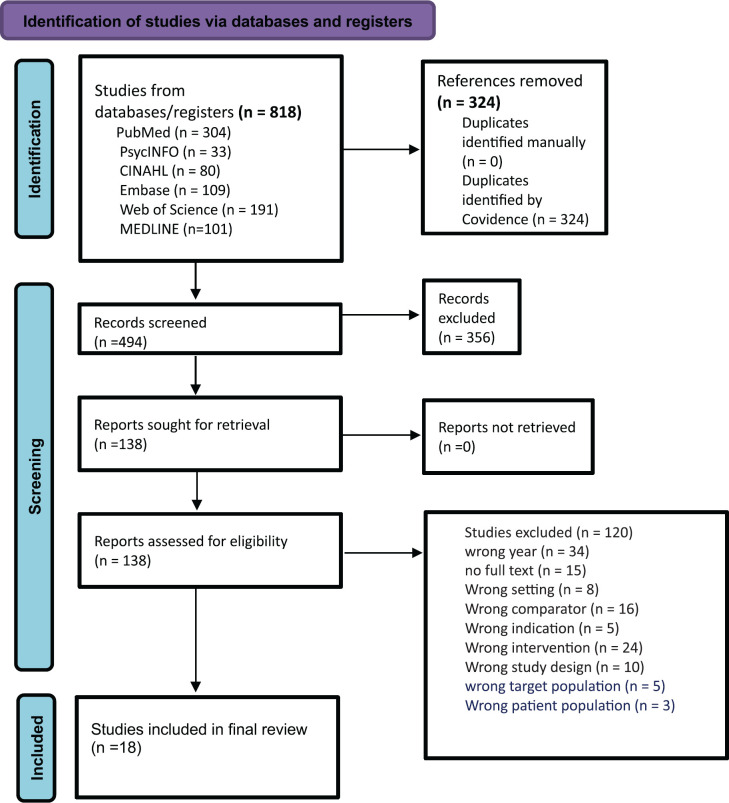
PRISMA flow chart.

A total of 818 studies were retrieved from the database search. After removing 324 duplicates, 494 studies remained for title and abstract screening. Of these, 356 studies were excluded, leaving 138 studies for full-text review. Subsequently, 120 studies were excluded for reasons such as publication year, lack of full text, inappropriate setting or wrong target population. Ultimately, 18 studies were included in this review (Figure 1).

### Study location

Thirteen (*n* = 13) of the included studies were conducted in the USA [12–24], while one study each was carried out in Canada [25], India [26], Malaysia [27], Mexico [28] and Nigeria [2]. According to World Bank [30], these studies were conducted in high-income countries, upper-middle income countries and lower-middle income countries.

### Study characteristics

Table 1 provides a detailed description of the characteristics of the included studies. The majority (*n* = 13) were published between 2012 and 2019, while five (*n* = 5) were published between 2020 and 2023. All included studies are primary research and comprise qualitative studies (*n* = 4), quantitative studies (*n* = 2), mixed-methods studies (*n* = 4), surveys (*n* = 5) and pilot studies (*n* = 2).

### Study participants

All participants in the included studies were female. The categories represented were African American (*n* = 5) [12, 13, 15, 19, 20], Chinese American (*n* = 2) [17, 21], Native American (*n* = 1) [16], Latin immigrant (*n* = 1) [24], Black and Latina women (*n* = 1) [23], Spanish-speaking Latinas (*n* = 1) [14] and immigrants (*n* = 1) [25].

#### Structure, strategy and delivery of peer education

Peer education in the studies included in this narrative review was implemented through a variety of structures, strategies and delivery systems (Table 2). The selection of peer leaders varied across studies: some were chosen through application and interview processes [25], while others emerged from community-led initiatives [18], community engagement and partnerships [22, 23] or volunteered for the role [26, 28]. In several studies, community health extension workers (CHWs) also served as peer leaders [17, 18, 24, 27]. Regardless of their selection method, all peer leaders received training to prepare them for their roles.

A range of educational materials and tools were utilised to support peer education. These included videos [13, 17, 18, 21], manuals [13, 24], PowerPoint presentations [14, 25] and demonstrations [16, 23, 26, 28].

The delivery methods were equally diverse. Group discussions [17, 21, 22, 23], virtual sessions [13, 27], focus groups [28], house-to-house visits [26] and teaching sessions [16, 22, 24] were all employed to engage participants and facilitate knowledge transfer. The studies demonstrate that peer education can be effectively adapted to different contexts through flexible selection processes, comprehensive training, varied educational resources and multiple delivery channels.

#### Impact of peer education in the prevention of breast cancer for community-based peer education interventions

Findings from multiple studies consistently demonstrate that peer education significantly enhances breast cancer awareness, knowledge, screening uptake, positive health behaviours and empowerment particularly in underserved communities (Table 3). For example, study by Ahmad *et al*. [24] reported that 48% of immigrant women in Canada who were previously under- or never-screened underwent mammograms following peer-led education. Similarly, findings from a study observed a dramatic increase in breast cancer screening rates, rising from 4.4% to 67.6% after the intervention [29]. In Latino communities, it was found that promotores (community-based peer educators) effectively increased awareness and screening by addressing linguistic and cultural barriers [14]. Findings from a study noted that 72% of women educated by lay breast health educators completed recommended screenings [23].

Culturally tailored peer-led interventions also yielded positive outcomes in Native American populations; findings reported an increase in the intention to undergo annual mammography from 81.1% to 94.6% [16]. Likewise, there was documented improvements in spiritual well-being, hope and patient-provider communication among African American breast cancer survivors who participated in peer support programs [15].

Regarding follow-up, studies by [23, 25, 26] monitored participants using phone calls, while [27] conducted a 4-month follow-up. These follow-up strategies likely contributed to the observed increases in screening uptake.

#### Challenges and facilitators associated with peer education approach

Despite promising outcomes, several challenges were identified across the studies. Rodriguez *et al* [25] reported tensions among peer educators as they struggled to balance their roles as both experts and community members. Baethge *et al* [13] found that although participants gained confidence in motivational interviewing (MI), mastering reflective listening and managing time constraints remained difficult. Barriers such as mistrust, lack of insurance and limited reach of educational resources were highlighted by [20, 24]. In rural India, [26] identified cultural taboos, low literacy levels and limited access to trained healthcare providers as significant obstacles. Similarly, [23] noted challenges in peer retention, transportation and screening attendance in the U.S., often linked to fear, undocumented status and systemic barriers.

Several facilitators enhanced the effectiveness of peer education programs. A key factor was cultural and linguistic alignment between educators and participants, which fostered trust and relatability, as reported by [14, 17]. Programs grounded in faith or cultural traditions, such as those described by [12, 22], benefited from community familiarity and moral authority. The use of culturally tailored tools including bilingual videos [21], storytelling and art [16] and breast models for demonstration [26] further supported effective learning. Additionally, comprehensive training and mentorship, as demonstrated by [24, 25], equipped peer educators with practical skills and structured educational materials, enhancing program delivery.

#### Factors influencing effectiveness of peer education

Community-based peer education interventions have proven highly effective in promoting breast cancer prevention among underserved women in diverse settings. A key factor was cultural and religious alignment between peer educators and participants, which fostered trust and helped overcome barriers like modesty, fatalism and stigma [15, 22, 23], Faith-based programs, such as mosque-centered education, notably increased women’s willingness and confidence to seek screening.

Language adaptation and literacy-sensitive approaches were also vital. [26] reached over 218,000 rural women using local languages, visual aids and storytelling, identifying numerous suspect cases. Similarly, [14, 25] found that materials tailored for low-literacy audiences improved understanding and retention.

Trust and community embeddedness consistently boosted participation. Engaging respected local figures, including breast cancer survivors and community health workers, led to substantial increases in screening uptake. For example, [29] reported cervical cancer screening rates rising from 3.2% to 67.6%, and clinical breast exams from 4.4% to 67.6%. Padela *et al* [23] found 72% mammography completion among women recommended by peer educators.

Effective training and ongoing mentorship enhanced peer educators’ knowledge and program adherence [17, 25]. Practical support, such as free screening, transport assistance and community-based sessions further increased participation, especially among low-income women [16, 29].

While cultural and behavioural factors are critical in shaping the effectiveness of peer education, system-level determinants also play a significant role in influencing outcomes. The availability and accessibility of early detection and diagnostic services, proximity of health facilities, cost of screening or treatment and trust in healthcare providers can all impact whether participants are able to act on the knowledge and motivation gained through peer-led interventions. Studies showed that even when peer education successfully increases awareness and intention to screen, structural barriers such as transportation challenges, financial constraints and limited healthcare infrastructure can limit actual uptake of services [23, 26, 28]. Integrating these system-level considerations into program design through strategies such as mobile clinics, subsidised screening, navigation support and partnerships with trusted healthcare providers can enhance the reach, uptake and sustainability of peer education interventions across diverse contexts.

#### Recommendations for designing peer education model

The studies included in this review offer several key recommendations for designing effective peer education models. First, lay health community-based programs should be integrated with strong collaborative partnerships within the community to enhance reach and acceptance [12, 23]. Peer education should incorporate multicomponent community education and navigation interventions to encourage positive health actions, often leveraging religious concepts to increase relevance and engagement [22, 27].

A train-the-trainer approach is recommended to build capacity, ensuring peer educators receive comprehensive training supported by appropriate educational materials and ongoing support [13, 14, 17, 24]. Additionally, delivering peer education through home visits or house-to-house outreach, coupled with the provision of preventive services at primary health care centers, can improve accessibility and uptake [29]. Overall, these recommendations emphasise the importance of community collaboration, culturally relevant content, capacity building and accessible delivery methods to maximise the impact of peer education programs.

## Discussion

This paper provides a comprehensive analysis of studies employing peer education as a tool for community breast cancer prevention. The rising incidence of breast cancer among women has driven researchers to develop educational interventions aimed at improving knowledge, beliefs and screening uptake.

The first objective of this review was to examine the structure, strategies and delivery methods of peer education programs in community settings. Findings show that peer educators primarily deliver training through group discussions [17, 21–23], supported by videos [13, 17, 18, 21], practical demonstrations [16, 23, 26, 28] and PowerPoint presentations [14, 25]. This aligns with [31], who reported interventions delivered either one-to-one or in groups. The results confirm the transformative role of peer education, especially among marginalised populations. Peer-led models effectively overcome cultural, linguistic and socioeconomic barriers by leveraging trusted community members. The grade or level of English used in the included studies was not explicitly stated; however, all studies employed culturally tailored interpersonal education approaches. This may be because most of the studies were conducted in the United States and Canada, which are Western and high-income settings with relatively homogeneous English language proficiency. In contrast, within the Low- and- Middle Income Country context such as Nigeria, the use of indigenous languages is often more effective for community-based interventions. This is supported by the findings of [32], who reported that during the critical COVID-19 period, health communication delivered in indigenous languages significantly enhanced the population’s understanding and engagement. Dramatic increases in screening rates from under 5% to over 60% in some studies [23, 29] demonstrate its impact. Beyond behaviour change, peer education fosters empowerment and agency among both educators and participants [15, 25]. Effectiveness is enhanced when educators share ethnic, faith or linguistic backgrounds with their audiences, acting as trusted messengers and cultural interpreters [14, 22]. Embedding sessions in familiar settings like churches, mosques or tribal centers and incorporating local art, food and storytelling also increases participation [12, 16]. These findings are supported by [33], who showed peer education improves health-related knowledge, and [34] who found significant gains in adolescent knowledge and behaviours through peer education.

Despite their promise, peer education interventions face challenges. Role confusion among educators in close-knit communities can cause strain, necessitating training that addresses boundary management and ongoing support [25]. Logistical barriers such as transportation and lack of insurance limit participants’ ability to act on education, risking stagnation at awareness without behaviour change. Cultural sensitivities may also restrict discussion of breast health, especially if educators are unprepared [24, 26]. These issues highlight the need for adaptable, context-specific approaches rather than one-size-fits-all models. Successful programs emphasise cultural competence, community ownership and structural support. Also, it is crucial to involve men in the intervention. Studies by [35] revealed that involving men in the intervention produces better results as they provide emotional and psychological support.

Structured and ongoing training, along with mentorship, are essential to maintain program fidelity and educator confidence [17, 25]. This is echoed by [36], who emphasised that peer education and counselling succeed only when organisational factors are well managed and cultural contexts respected. Attention to program structure, management, training, supervision, support, monitoring and retention is critical for effective peer education initiatives. Community collaboration, culturally relevant content, capacity building and accessible delivery methods should be carefully considered when designing peer education models to maximise their impact. Co-creation of intervention materials with community involvement is a collaborative and democratic approach that recognises both community members and professionals as equal partners in developing community-based peer education [37]. This approach has been applied across a variety of healthcare settings and with diverse patient populations to design person-centered interventions that respond to patient-identified needs. Its application in community-based education is similarly expected to enhance relevance, acceptability and effectiveness.

Although most of the studies included in this review were conducted in high- or middle-income countries, their findings can be adapted to lower-resource settings, such as Nigeria. Translation of these interventions requires culturally relevant content, accessible delivery methods and strong community collaboration. Additionally, local health system constraints, resource limitations and factors such as availability of early detection services, transportation challenges and trust in healthcare providers must be considered to ensure program feasibility, acceptability and effectiveness. Incorporating these adaptations can help bridge the gap between evidence from higher-income settings and practical implementation in Nigerian communities, enhancing the relevance and impact of community peer education interventions.

### Implications for practice and recommendations

Based on the findings of this review, the following recommendations are proposed:

1. Integrate peer education into community structures. Leveraging women’s groups, religious networks, cooperatives and other established community organisations enhances credibility and participation [23, 25]. Implementation should engage community-embedded peer educators, CHWs, local survivor-led groups, faith-based educators, and existing health structures.
2. Provide structured training, mentorship and supervision. Clear guidance and support reduce role ambiguity and strengthen program delivery [17, 25]. Training should be delivered by program supervisors, research teams, health-education institutions and advisoryboard-guided trainers.
3. Mitigate logistical and cultural barriers. Transportation challenges, stigma, and other contextual barriers [24, 26] can be addressed through flexible, community-based program designs. CHWs, local health facilities and community educators can manage operational challenges, while policymakers address financial and systemic constraints.
4. Adapt evidence-based models to local contexts. Successful interventions from high-income settings should be tailored to align with local health systems and cultural norms. Researchers and program designers should collaborate with local health authorities, community stakeholders and grassroots health workers to ensure feasibility and acceptability.

## Limitations

This review was limited by the predominance of studies conducted in high-income countries and methodological constraints such as small sample sizes and cross-sectional designs. These limitations reduce the direct generalizability of findings to low-resource settings. Nonetheless, by synthesising global findings and contextualising their relevance for Sub-Saharan Africa, this review contributes practical insights for adapting peer education locally. Future research should prioritise longitudinal and mixed-method designs in African and other resource-constrained settings to evaluate sustainability, scalability and long-term health outcomes.

## Conclusion

This review adopted a narrative approach to examine the role of community-based peer education in breast cancer prevention. Through a systematic search of six databases, studies published between 2010 and 2025 were identified and screened using strict inclusion and exclusion criteria, ensuring a clear focus on peer-led interventions in culturally diverse and low-resource community settings. By incorporating qualitative, quantitative and mixed-methods studies, the review captured a nuanced understanding of strategies, challenges and contextual factors influencing intervention outcomes. Data extraction and thematic analysis enabled the identification of recurring patterns and effective practices. Findings highlight peer education as a culturally adaptable, cost-effective and community-driven approach to improving breast cancer awareness and early detection, particularly among underserved populations. This review also emphasises the importance of system-level considerations, including access to early detection and diagnostic services, proximity of healthcare facilities, affordability and trust in healthcare providers, as these factors influence the success of peer-led interventions. Based on the evidence, several recommendations emerge for policymakers and healthcare professionals: integrating peer programs into primary care and community health structures, establishing structured training and mentorship frameworks for peer educators, providing mechanisms to sustain engagement over time and ensuring adequate funding to maintain program reach and quality. Collectively, these strategies can enhance the effectiveness, scalability and sustainability of community-based peer education programs, ultimately contributing to improved breast cancer outcomes in diverse populations.

## Author contributions

Conceptualisation and design: ROO, CMN, GOA, IOO, OA. Literature search: OA, ROO, CMN, IOO, GOA. Manuscript writing and editing: ROO, GOA, FAA, DA, SA, HAG, DA, IOO, CMN. Data Extraction and Synthesis: ROO, GOA, FAA, DA, SA, HAG, DA. Supervision: CMN, OA. Final approval of manuscript: All authors.

## Conflicts of interest

The authors declared no conflicts of interest.

## Funding

There was no external funding available for this study.

## Figures and Tables

**Table 1.** Geographic distribution, objectives and focus areas for community-based peer education interventions.

Author/Year	Location	Objective	Focus
Ahmad *et al* (2016)	Toronto, Ontario, Canada.	Explore experiences of peer leaders who worked for the Cancer Awareness: Ready for Education and Screening (CARES) project to promote awareness, knowledge and uptake of breast cancer and cervical cancer screening among under/never screened women	Cervical and breast cancer
Allicock *et al* (2017)	USA - Rural North Carolina	Describe the training and evaluation of community coaches and guides using the Peer Connect program for African American breast cancer survivors/care givers	Breast cancer
Almeida *et al* (2021)	USA	To develop comprehensive culturally acceptable materials for community health educators (promotores)-led hereditary breast cancer education programs for Spanish-speaking Latinas	Breast cancer and genetics
Anderson-Lewis 2012	USA	Provide insight into breast cancer information acquisition experiences of African American women	Breast cancer
Ashing-Giwa *et al* (2012)	USA	Examine the impact of support groups among African American breast cancer survivors	Breast cancer survivors
Chigbu *et al* (2017)	Southeast Nigeria	Determine the impact of trained community health educators on the uptake of cervical and breast cancer screening and HPV vaccination in rural communities in Southeast Nigeria	Cervical and breast cancer
Chilton *et al* (2013)	Southeast Texas, USA	Raise awareness of native American women to breast cancer	Breast cancer
Gu *et al* (2019)	United States	Evaluate Chinese American community health workers training and implementation program on mammography	Breast cancer
Hamlish *et al* (2023)	USA	Implemented a virtual learning collaborative to build community capacity to deliver breast cancer survivorship navigation	Breast cancer
Hanson *et al* (2013)	USA	Examine whether peer support intervention can help people with persistent and serious illness	Advanced care
Hempstead *et al* (2018)	USA	Examination of peer educator interventions to determine whether the intervention increases knowledge; assessment of whether peer educators intend to share information and with whom; and evaluation of any increase in knowledge among friends and family because of the information received.	Breast cancer
Maxwell *et al* (2011)	Los Angeles., Washington, California	Evaluate the feasibility of training Chinese American lay educators to recruit and deliver breast health educational sessions	Breast health
Padela *et al* (2018)	USA	To describe the design of and participant-level outcomes related to a religiously tailored, peer-led group education program aimed at enhancing Muslim women’s mammography intention	Mammography screening
Rodriguez *et al* (2020)	South Florida, USA	Develop a context-specific, culturally appropriate training intervention for South Florida community health workers to educate Latinx immigrant farmworkers on breast cancer and early detection	Breast cancer
Sangwan *et al* (2023)	India	To strengthen breast cancer screening program using BSE strategy and adopting a referral mechanism for the diagnosis and treatment of suspect cases	BSE
Schliemann *et al* (2023)	Malaysia	Evaluation of intervention that used mHealth and community health workers to educate women about breast cancer screening and navigate them to clinical breast examination services in the context of COVID-19 pandemic	Breast cancer
Soto-Perez-De-Celis *et al* (2017)	Mexico.	Assessed the feasibility of a breast cancer educational program for adolescents attending middle school in rural Mexican community	Breast cancer
Torres *et al* (2017)	USA	Training and supporting lay members who serve as breast health educators to increase breast self-awareness and knowledge of breast health, screening mammography, screening guidelines and the importance of early detection, while also reducing barriers to accessing breast screening services.	Breast cancer screening

**Table 2.** Study design, target groups and delivery approaches for community-based peer education interventions.

S/N	Author & Year (Country)	Study design	Target population	Peer education structure (Who were the peers) e. g trained female community health volunteers	Delivery methods & strategies
1	Ahmad *et al* (2016) (Canada)	Qualitative study using focus groups (3 FGs with 14 participants).	Recent immigrant women aged 30–50 from ethnic minority and low-income backgrounds.	Peer leaders (all immigrant women) were selected through applications and interviews. Criteria: bilingual, interest in women’s health, public speaking, teamwork. Received 3-day training including communication, facilitation, adult learning, health topics, cultural sensitivity and ongoing mentorship.	Multilingual educational group sessions with visuals and PowerPoint, screening accompaniment, phone follow-ups. Sessions conducted in 20 languages at over 60 community sites.
2.	Allicock *et al* (2017) (USA)	Mixed method (Pilot study)	African Americans breast cancer survivors and caregivers in North Carolina	Train-the-trainer model using Peer Connect - Community Coaches were trained to train Guides.	3-day DVD and manual-based Motivational Interviewing (MI) training, Guide Gatherings (monthly), telephone and in-person peer support sessions, local recruitment via flyers, word-of-mouth and emails to existing cancer related listservs.
3	Almeida *et al* (2021) (USA)	A mixed methods study using qualitative and quantitative analyses of multiple discussion.	Spanish-speaking Latinas	Recruitment of participants was led by The Latino Cancer Institute (TLCI). The study included two types of participants: promoters (peer educators) and community members:	Power point presentation and surveys meetings lasted between 60 and 90 min, was audio-recorded, conducted in Spanish and moderated by two bilingual members of the research team. Questionnaire was also distributed to the community members.
4	Anderson-Lewis *et al* (2012) (USA)	Qualitative – Focus Groups	African American women aged 35+, not previously diagnosed with breast cancer	Four trained African American women as Community Health Advisors/Research Partners (CHARPs) in their communities	Focus groups; peer-led discussions; culturally tailored interpersonal education through churches, family reunions and community spaces
5	Ashing-Giwa *et al* (2012) (USA)	Qualitative (focus groups)	African American breast cancer survivors (BCSs)	Peer-led support groups formed via the African American Breast Cancer Coalition (AABCC). Groups led by trained survivors and community advocates, culturally embedded in African American norms and values.	Group-based support meetings conducted in churches and community centers. Activities included emotional sharing, faith-based prayer, information exchange, navigation assistance, advocacy training and outreach events.
6	Chigbu *et al* (2017)	Prospective population-based intervention	Community Health Educators on Cancer Prevention (CHECPs)	Use of existing community health infrastructure and trusted local nurses. Training provided to CHECPs ensured the quality and consistency of information. Proximity of health facilities (within walking distance) facilitated access to services	House to house cervical and breast cancer prevention education by the trained CHECPs
7	Chilton *et al* (2013) (USA)	Quantitative (Pre-post survey design)	Native American women aged 36–69	Community-led initiative, designed with tribal leaders, included community liaison and breast cancer survivor sharing personal story.	One-day culturally tailored event: free mammography, breast health games, bead activities representing tumour sizes, exercise (Nia technique), cooking demo with traditional foods, art projects, transportation provided, bilingual support.
8	Gu *et al* (2019)(USA)	Mixed-method process evaluation of a randomised controlled trial	Chinese American women aged 40+, mostly non-adherent to mammography screening	26 CHWs trained in a one-time 4-h session (video-based, discussion guides, role play). Recruited from community-based organisations. Used the Health Belief Model.	Small-group sessions (5–11 women), video screening, structured discussion in Chinese, take-home DVD and local screening resource guide.
9	Hamlish *et al* (2023) (USA)	Quantitative – Pre-post intervention survey	Community health workers and patient navigators (98% female)	Participants (CHWs and navigators) were trained in a 14-week virtual learning collaborative (VLC) to enhance survivorship navigation	Weekly 60-min virtual sessions with didactics and case presentations; VLC format to build knowledge and navigation capacity
10	Hanson *et al* (2013) USA	A community-based participatory research approach was conducted	African Americans facing advanced cancer and serious illness and African American churches and community organisations	All participants were African American women and 25 volunteers completed training as lay health advisors	Support intervention included a combination of training and community engagement. Volunteers received a 3-h team member training or a full day-long leader training and also six to ten volunteers were trained to work collectively in providing ongoing support to individuals with serious illness.
11	Hempstead *et al* (2018)	Culturally tailored for African American women, addressing mistrust and barriers.	African American women	Peer educators called Community Empowerment Partners (CEPs) were recruited by the Cierra Sister director and were trained in a one day 6 h training	CEP toolkit was used in facilitating the breast cancer education community workshops
12	Maxwell *et al* (2011)	A pilot, community-based intervention	Chinese American women	A trained Chinese American woman who served as lay health educators. They were recruited to conduct small-group sessions after attending a 3-h training conducted in Chinese and responsible for showing a culturally tailored video promoting mammography screening, facilitating discussions about barriers and strategies and distributing educational materials.	Small group session as the primary delivery method and sessions were conducted at community settings such as churches, community-based organisations and private residences. a culturally tailored, 18-min soap-opera style video in Mandarin
13	Padela *et al* (2018)	A community-engaged approach and mixed methods	Muslim women	Potential peer educators were identified from mosques and Community Advisory Board (CAB) networks. Screening was done through phone call to assess their eligibility and interest in participating. Informed consent was obtained and participants attended a two-session training course	Peer educators facilitated group discussion while guest lecturers taught about mammography, conveyed Islamic teachings about health and provided resources for accessing mammography
14	Rodriguez *et al* (2020) South Florida (USA)	Formative research strategy Guided by the principles of Community based participatory research (CBPR) study and Qualitative data	Study was conducted among Community health worker (CHW) and Latinx immigrants farmworkers	CHW training curriculum was first piloted with 14 Spanish-speaking CHWs who work outside of the farmworker community. In June 2019, two formal training sessions (one in English, one in Spanish) were administered to eight CHW employees of Community Health of South Florida. Three-hour training were structured around the manual chapters	Trainees were given copies of the training manual to follow during the sessions, flipbooks to use in role-playing activities. pre- and post-tests were administered to assess CHW knowledge gains and training effectiveness. The pre-/post-training test isa 43-question survey adapted from Keating *et al*. to assess breast cancer knowledge immediately before and after training sessions.
15	Sangwan *et al* (2023) (India)	Community-based intervention study (2017–2022)	Women aged 30–65, plus primary healthcare providers (MOs, ANMs, ASHAs, AWWs)	Training cascade: MOs → ANMs/LHVs → ASHAs and AWWs. Peer volunteers were also engaged. Focused on BSE using culturally relevant materials.	House-to-house visits, group meetings, dummy breast model demonstrations, videos, booklets, phone follow-up.
16	Schliemann *et al* (2023)	Female CHWs for norms; male CHWs for decision-makers; tailored materials.	COVID-19, low online engagement, demographic disparities.	High mobile use, trusted CHWs, supportive clinic, free services.	Scale up CHWs and mHealth, use trusted institutions, tailor content, improve digital engagement, validate beyond pandemic
17	Soto-Perez-De-Celis *et al* (2017) (Mexico)	Pilot study with four months follow up (of students and relatives)	Female adolescent students and their older female relatives	School based educational sessions led by pedagogy students, medical students, a breast cancer survivor with supervision from two Oncologists (ES, YC) who were available to answer questions.	Five 40 min educational sessions -breast cancer information -a survivor story -guided breast self examination -critical thinking collage -role playing activity Four groups of 25 to 30 students each in different classrooms, took place in a no school day with small gifts, breaks, lunch and 4 months follow-up
18	Torres *et al* (2017) (USA)	Qualitative summative evaluation (with descriptive stats)	Uninsured and underinsured women aged 25+ (majority Black and Latina)	23 trained Lay Breast Health Educators (LBHEs), selected via community partnerships. Some were breast cancer survivors or bilingual. Also involved Public Health students for field training support.	Education: Group-based community sessions, ~10 minutes each in settings like clinics, faith-based centers, food banks and health fairs. Navigation: one-on-one assistance, appointment scheduling, interpretation, transport support, follow-up calls.

**Table 3.** Outcomes, contextual influences, implementation barriers and enablers and key recommendations.

S/N	Author & Year	Outcomes measured (e.g. knowledge, attitude, screening behaviour	Cultural/contextual factors	Implementation challenges	Implementation facilitators	Recommendations
1.	Ahmad *et al* (2016)	Among under-/never-screened women: 43% went for Pap tests, 48% had mammograms. Peer leaders reported increased confidence, self-care and community health advocacy. Empowerment also reported.	Multilingual delivery addressed language barriers. Cultural beliefs (e.g. fatalism, myths), recent immigrant experiences and ethnic community norms influenced participation and role identity	Navigating dual roles as ‘expert’ and ‘peer.’ Tensions between science and cultural myths and between professional versus personal boundaries.	Trust built within ethno-cultural communities, shared language, lived immigrant experience, institutional affiliation and structured mentorship.	Training should incorporate role identity negotiation, professional development and contextual sensitivity. Peer programs should support dual role navigation and offer structured mentorship.
2	Allicock *et al* (2017)	Feasibility, MI fidelity and acceptability of peer training; increased self-efficacy to use MI; MI-appropriate responses increase from pre- to post-test	Focused on psychosocial needs Matched participants by race, gender and survivor/caregiver role Trusted community organisations involved	Modest success with MI fidelity Difficulty mastering reflective listening Time constraints and limited group activities	Train-the-trainer approach with community ownership Manual/DVD structure Prior relationships with trusted local organisations Motivation among participants	More MI practice and technical support Conduct mock trainings Assess long-term implementation fidelity
3	Almeida *et al* (2021)	Promotores-based BC awareness, screening and navigation programs for the US Latino community have been shown to be effective.	The use of peer educators (promotores) is very effective in serving as a bridge between underserved Latino communities and the healthcare system. Sharing a similar sociocultural identity ethnicity, language, socioeconomic status and health care experiences) with other community members provides promotores a deep and unique understanding of the community’s belief and value system. Specifically, evidence supports the cost-effectiveness of promotores-led educational interventions to increase cancer screening in the latino community.	Both promotores and community members reported concerns about barriers to access health care and health education among Latinos. Regarding cultural barriers, the most common were misconceptions (e.g. beliefs about mammography causing cancer or being unbearably painful), fatalistic views, mistrust of health care providers and cultural values, such as prioritising their family’s health over their own. One promotors said‘as women and mothers, many times we first pay attention to the children, the husband or the rest of the family.	Use of culturally similar promotores; collaboration with community leaders and CAB; culturally tailored materials in Spanish; narrative storytelling; support from a trusted Latino health organisation.	Study strongly suggests that educational materials on complex concepts, such as genetics and cancer, can be effectively created as a collaborative effort between researchers and a strong and dedicated CBO to engage the community.
4.	Anderson-Lewis *et al* (2012)	Improved breast cancer knowledge pathways; preference for interpersonal sources (physicians and peers); low preference for Internet	Strong faith-based orientation; language complexity concerns; community-based trust and support networks; digital divide	Information overload online; lack of trusted community messengers; low reach of educational resources	CHARPs were trusted and embedded in communities; church/family gathering access points; relatable language	Use CHARPs; simplify health messages; promote outreach through culturally familiar spaces; train lay educators
5.	Ashing-Giwa *et al* (2012)	Positive impact on hope, outlook, spirituality, symptom management and navigation. Improved patient-provider relationships, family life and coping behaviours.	Strong influence of spirituality, language and shared cultural experience. Faith-based community norms, family structure and reluctance toward mainstream services.	Underrepresentation in traditional support services; cultural mismatch in mainstream care; secrecy/taboo around cancer.	Trust in culturally aligned peers; church-based settings; advocacy focus; deep emotional connection and spiritual grounding.	Develop culturally tailored peer support models; integrate spiritual, emotional and community advocacy components into survivorship care. Engage African American survivors in program design.
6	Chigbu *et al* (2017)	Post-intervention, 67.6% of women underwent cervical cancer screening (up from 3.2% pre-intervention) and 67.6% received CBE (up from 4.4% pre-intervention). Among eligible children, HPV vaccination uptake increased from 0.9% to 33.2%	The intervention targeted rural, underserved communities with low cancer prevention awareness, using trusted community nurses and local-language home visits to overcome cultural barriers	Low baseline awareness and uptake of cancer prevention services. Financial barrier for HPV vaccination, which required a fee (about US$12), while other services were free	Use of existing community health infrastructure and trusted local nurses. Training provided to CHECPs ensured quality and consistency of information. Proximity of health facilities (within walking distance) facilitated access to services	Community-based, peer-led education significantly improves cancer prevention uptake Scale-up efforts should focus on removing financial barriers (e.g. free HPV vaccination) and leveraging policy change for broader implementation
7	Chilton *et al* (2013)	Increase knowledge of breast cancer risks (e.g. age and nulliparity) Increase belief in early detection Increase intention to get annual mammograms (81.1%â€“94.6%)	Native art, storytelling, cooking and spiritual alignment Community-based participatory research model Distrust of mainstream care	Fear of diagnosis Limited prior positive screening experiences Transportation needs Low historical screening rates	Culturally tailored programme, inclusion of Native foods and creative arts, trusted community members leading, transportation provided, free screening	Promote CBPR-based approaches, include cultural art and food, address fears directly, improve access/logistics, support peer-led education through storytelling and tribal leadership
8.	Gu *et al* (2019) (USA)	Significant improvement in CHW knowledge (*p* < 0.01). Implementation fidelity: 10/13 CHWs correctly addressed >3 of 5 benefit items; 11/13 addressed >5 of 9 misconceptions.	Language barriers, cultural misconceptions (e.g. fate, stress, small breast myth), trust in peer-led discussion helped challenge beliefs.	One-time training led to variable fidelity. Some CHWs added personal opinions or misinformation despite training.	Community-based organisation partnerships, CHWs’ cultural/linguistic similarity with participants, use of visual aids and simplified guides.	Ongoing or booster trainings are needed. Structured discussion tools aid fidelity.
9.	Hamlish *et al* (2023	24% increase in navigator self-efficacy; major gains in ability to consult, explain trials and connect survivors to resources; no behavioural change	Served urban/suburban diverse communities; predominantly female and racially mixed group	No observed behaviour changes despite improved self-efficacy; navigation still offered without structured training	Virtual format allowed wide participation; VLC encouraged sharing and learning from real cases; improved knowledge	Invest in VLCs to build capacity; recognise and reimburse CHWs for survivorship support roles; bridge care beyond diagnosis
10	Hanson *et al* (2013)	This community-based participatory research project, conducted in two phases over a 5-year period, demonstrates the feasibility and initial impact of peer support for African Americans facing advanced cancer and other serious illnesses.	The evaluation revealed important barriers to the lay health advisor model for support of those with serious illness. Even in their applications, volunteers anticipated challenges, inadequate knowledge of cancer treatment, fear of being with someone who might die, concern about the required time commitment, difficulty controlling emotions, uncertainty about how to address patientâ€“family conflicts and a need to set limits when patients’ needs exceeded the volunteers’ ability to respond.	Patient privacy barriers in the community made limiting peer support nearly impossible as people did not always reveal their diagnosis or cancer stage. In addition, many volunteers rejected limiting the support team model to cancer. Although the marked needs Of cancer patients were clear to them, volunteer teams also saw the relevance of their support to people with other types of serious illness. In the spirit of community-based participatory research, investigators modified the protocol in response to these concerns.	Use of existing social networks; church-based adoption; team-based peer model; strong academicâ€“community partnership; culturally grounded support through spirituality and emotional care.	Further research should test the efficacy and maintenance of this intervention and its ability to be exported to other settings.
11	Hempstead *et al* (2018)	Improved knowledge and intention to share info.	Culturally tailored for African-American women, addressing mistrust and barriers.	Mistrust, lack of insurance, discrimination.	trusted partners, respected peer educators, relevant materials.	Peer programs are effective and support wide community knowledge sharing.
13	Maxwell *et al* (2011)	Results suggest that Chinese American volunteers are willing to attend training, to recruit women and to conduct small-group sessions, although only nine out of a total of 30 trained community volunteers actually conducted a session due to the short study duration.	Breast health tea time workshops’ were conducted at churches, community-based organisations and private residences. Each session started with watching an 18-minute cultural video in Mandarin or Cantonese.	All group attendees were foreign-born. Less than half had some college or higher level of education, 63% reported speaking English poorly or not at all, and only 58% had health insurance. Forty-three percent of the women had never had a mammogram. Other studies also suggest that many Asian American women do not value or are not familiar with the concept of preventive health care	volunteer participation, community organisation and private setting. use of culturally tailored video format in community settings	Given that this pilot study did not include a comparison group, future studies should more rigorously test the impact of this promising strategy.
13	Padela *et al* (2018)	Primary: Mammography intention, likelihood, confidence Secondary: Actual mammogram receipt (6-month and 1-year follow-up)	Addressed modesty, fatalism, fear and religious beliefs Delivered by ethnically/religiously concordant educators Embedded within faith-based identity and mosque life	Small, English-speaking, religiously inclined sample Generalisability limited to mosque-going women	Strong community engagement via advisory board Mosque support; religious scholar involvement; culturally relevant materials	Scale religiously tailored, mosque-based education Use faith-aligned messaging and peer models Target religion-related barriers in similar underserved groups
14	Rodriguez *et al* (2020)	The formative CBPR findings informed the adaptation of breast cancer educational materials, originally developed for health promoters in Mexico, to the specific context of this Latinx farmworker community in the U.S. with high reported late-stage breast cancer diagnosis rates. Specifically, the community priorities and CHW knowledge gaps that we identified in South Florida were key in guiding the design of the overall training curriculum and material dissemination strategy.	Strong faith-based orientation; language complexity concerns; community-based trust and support networks; digital divide.	Information overload online; lack of trusted community messengers; low reach of educational resources.	Community Engagement and Participation, partnership and also training of CHWs with practical skills	Involvement of community stakeholders and intended end-users in the design of a CHW breast cancer curriculum and educational materials resulted in a training intervention that effectively targeted specific needs and knowledge gaps and a portfolio of dissemination materials that are relevant, appropriate and tailored to this marginalised population. Should be encouraged
15	Sangwan *et al* (2023) (India)	From <1% BSE awareness to 218,978 women trained. 745 suspect cases identified (332 due to painless lumps). 16 confirmed cases. 824 healthcare workers trained.	Low literacy, cultural hesitance, poor attitudes toward BSE, lack of prior screening experience.	Cultural taboos, limited access to follow-up care, lack of training among FHWs, time constraints.	Use of visual tools, community volunteer support, repeated follow-ups, engagement of local health departments.	Promote BSE as an affordable tool. Train grassroots workers thoroughly. Use community mobilisation. Model can be replicated in other regions.
16	Schliemann *et al* (2023)	CBE uptake, screening intention, acceptability, satisfaction.	Female CHWs for norms; male CHWs for decision-makers; tailored materials.	COVID-19, low online engagement, demographic disparities.	High mobile use, trusted CHWs, supportive clinic, free services.	Scale up CHWs and mHealth, use trusted institutions, tailor content, improve digital engagement, validate beyond pandemic
17	Soto-Perez-De-Celis *et al* (2017)	Increased breast cancer knowledge from baseline to 4 months follow up. Students: 63%â€“82% Older relatives: 55%â€“61% (Intergenerational transmission of knowledge to female relatives).	Strong schoolâ€“family links cultural norms (female-only) Rural Mexican context with limited breast health exposure	Self-selection bias Male adolescents excluded Relative questionnaire were unsupervised Delivered by outside team, not local educators	Sessions culturally adapted Integrated into school calendar Team included survivor/educators Support from teachers Follow-up maintained	Focusing on third grade students, creating breast cancer educational programs for males, and implementing the program biannually.
18	Torres *et al* (2017)	Increased breast cancer knowledge and screening uptake. 72% of those recommended got screened.	Cultural tailoring to Black and Latina communities. Use of bilingual educators, addressing myths (e.g. only symptomatic women need screening), religion, language barriers, stigma around cancer and undocumented status.	Peer retention due to personal/family/health issues; no-shows for mammograms due to fear, transport, legal status concerns, changes in insurance; difficulty navigating women through full continuum due to structural/systemic barriers.	Use of community-trusted peers (survivors, faith leaders); bilingual education; stakeholder partnerships; stipends for peers; education + navigation; accessible screening sites; phone reminders and follow-ups.	Continue culturally tailored outreach; partner with community orgs; diversify outreach channels (e.g. churches); use testimonials.
